# New association of bone morphogenetic protein 4 concentrations with fat distribution in obesity and Exenatide intervention on it

**DOI:** 10.1186/s12944-017-0462-1

**Published:** 2017-04-04

**Authors:** Xingchun Wang, Jiaqi Chen, Liang Li, Cui Ling Zhu, Jingyang Gao, Sharvan Rampersad, Le Bu, Shen Qu

**Affiliations:** 1 Department of Endocrinology and Metabolism, Shanghai Tenth People’s Hospital of Tongji University, School of Medicine, Tongji University, 301 Middle Yanchang Road, Shanghai, 200072 China; 2 Department of Endocrinology and Metabolism, Suzhou Municipal Hospital, Suzhou, Jiangsu Province, 215000 China

**Keywords:** Obesity, Exenatide, Bone morphogenetic protein 4, Fat distribution

## Abstract

**Background:**

Bone morphogenetic protein 4 (BMP-4) has been proven to regulate white adipogensis. We aimed to demonstrate the correlation of BMP-4 with fat distribution and Exenatide treatment on it.

**Methods:**

We enrolled 69 obese patients. Anthropometric and metabolic indexes were collected. Fat distribution was measured by dual-energy X-ray absorptiometry. BPM-4 levels were assessed using enzyme-link immunosorbent assay kit. 30 obese patients were treated with Exenatide twice a day. Change in body weight, metabolic-related indices and BPM-4 levels were evaluated after 18 weeks.

**Results:**

1) The mean(±SD) BMP-4 levels were 763.98 ± 324.11 pg/ml in the obese. BPM-4 levels were significantly positively correlated with estimated visceral adipose tissue mass in all subjects and also in females (*r* = 0.377, *r* = 0.625, respectively,all *P* < 0.05). BPM-4 levels were also significantly positively correlated with body mass index, hip circumference and total fat% in females (*r* = 0.375,*r* = 0.429,*r* = 0.493,respectively, all *P* < 0.05). BPM-4 levels were negatively correlated with total cholesterol(TC) in all subjects and males also (*r* = −0.373,*r* = −0.332,respectively, all *P* < 0.05). BPM-4 levels were also significantly positively correlated with free triiodothyronine in males (*r* = 0.441, *P* < 0.05). 3) Multivariate analyses showed that TC was risk factor of BMP-4 concentration in males and Est.VAT Area was risk factor of BMP-4 levels in females. 4) BMP-4 levels were significantly higher in the obesity with slightly increased thyroid stimulating hormone(TSH) than the obesity without slightly increased TSH (902.08 ± 354.74 pg/ml vs. 720.24 ± 306.41 pg/ml, *P* < 0.05). 5) Exenatide treatment leads to a significant decreased in BMP-4 from 860.05 ± 352.65 pg/ml to 649.44 + 277.49 pg/ml independent of weight loss(*P* < 0.05).

**Conclusion:**

BMP-4 levels were associated with the visceral adipose tissue and may play a certain role in fat distribution and subclinical hypothyroidism in obesity. Exenatide treatment reduced BMP-4 levels independent of weight loss.

**Trial registration:**

Clinicaltrials.gov Identifier: NCT02118376, Registered 16 April.

## Background

Obesity is a worldwide health problem with increasing prevalencecharacterized by increase in adipocytes [1]. Obese patients are prone to developing insulin resistance and type-2 diabetes (T2DM). The increasing number and size of mature adipocytes results in obesity [2]. Adipose tissue is classified as white adipose tissue (WAT) and brown adipose tissue (BAT). The increased adipocyte plays a major role in the related complications in obese patients as WAT is primary site of triglyceride storage [3]. Fat accumulates in different ectopic sites, including visceral adipose tissue, which is responsible for the obesity related metabolic consequence [4]. However, excess fat stored in the subcutaneous has a protective effect against these complications [5].

The bone morphogenetic proteins (BMPs) have been shown to recruit adipose precursor cells into the adipose lineage [6]. Bone morphogenetic protein 4(BMP-4) is a growth factor of the transforming growth factor-β superfamily that is related to white adipogenesis [7, 8]. Recent data has certified that BMP-4 plays a role in obesity as BMP-4 is an integral feedback regulator of both white and beige adipogenic commitment and differentiation [9]. BMP-4 signaling is involved in the common pathophysiology of obesity and abnormal glucose metabolism [10]. Son et al. [11] found that serum BMP-4 levels in non-diabetic patients with obesity and metabolic syndrome were significantly increased. Glucagon-like peptide-1 (GLP-1) receptor agonist has been shown to be effective in decreasing body weight and improving fat distribution. The mechanism of its action is activation of GLP-1R in the hypothalamus dephosphorylates adenosine monophosphate-activated protein kinase(AMPK) and stimulates BAT thermogenesis and energy expenditure [12].

However, the association of BMP-4 levels with metabolic parameters and fat distribution, and the interaction of GLP-1 and BMP-4 has not been completely expounded. Therefore, in this study, we aimed to investigate the association of serum BMP-4 levels with fat distribution and the effect of GLP-1 receptor agonist(Exenatide) treatment on it in obese patients.

## Methods

### Patients and exenatide intervention trial

Sixty-nine obese Chinese patients from the outpatient of the Endocrinology and Metabolism department at the Shanghai Tenth People’s Hospital, who all provided informed consent, were included in this study (aged from 18 to 68 years old). Obesity was defined by the criteria that the body mass index (BMI) was over 28 kg/m^2^. The exclusion criteria were as follows: 1) clinical or laboratory evidence of impaired cardiac, liver and renal function. 2) severe systemic diseases (e.g., cancer and heart failure), and 3) taking medications known to affect body weight. 4) a history of severe gastrointestinal diseases. Among them, a total of 30 obese patients were recruited for Exenatide treatment. They received 5 mg exenatide twice a day for the first 4 weeks, which was then increased to 10 mg twice a day for the remaining 14 weeks. Before and after the 18-week treatment, their metabolic parameters were tested. This study was approved by the ethics committee of Shanghai Tenth People’s Hospital. The Clinical Trials registration Number is NCT02118376.

### Anthropometry

Weight was measured with light clothes and without shoes in all subjects. BMI was calculated as weight (kg)/(height^2^) (m^2^). Neck circumference (NC) was measured horizontally at the upper margin of laryngeal prominence with head erect and eyes facing forward. Waist circumference (WC) was measured at the point midway between the lower rib margin and the superior iliac crest along the mid-axillary line using a measuring tape. Hip circumference (HC) was measured at the maximum point around the buttocks. The waist to hip ratio (WHR) was calculated as the WC divided by HC. After the subjects rested for 10 min, blood pressure (BP) (systolic pressure (SP) and diastolic pressure (DP)) was measured while they were seated. BP was measured twice and the average value was used for analysis.

### Biochemistry and fat content

Blood samples were collected after all subjects were fasted for 12 h. These biochemical parameters included alanine transaminase (ALT), aspartate aminotransferase(AST), fasting plasma glucose (FPG), fasting insulin (FINS), C-peptide, glycated hemoglobin A1C (HbA1C), total cholesterol (TC), triglyceride (TG), high density lipoprotein cholesterol (HDL-C), low density lipoprotein cholesterol (LDL-C), C reactive protein (CRP), free fatty acids (FFA), uric acid (UA), free triiodothyronine(FT3), free thyroxine (FT4) and thyroid stimulating hormone(TSH). Insulin resistance was assessed using Homoeostasis Model of Insulin Resistance(HOMA-IR) calculated by fasting plasma glucose (mmol/L) × fasting plasma insulin (uU/mL)/22.5 [13]. Body fat mass of different body parts were measured with high accuracy by dual-energy X-ray absorptiometry (DEXA) (Hologic QDR4500, USA). We selected the data from DEXA including %fat trunk/%fat legs, total fat mass, trunkfat%, estimated visceral adipose tissue Area (Est. VAT Area), total fat%, trunk fat mass, trunk/limb fat mass ratio.

### BMP-4 measurement

Blood samples were collected after all subjects were fasted for 12 h. Serum BMP-4 levels were measured using a Quantikine human BMP-4 ELISA Kit (abcam, Catalog Number ab99982, Cambridge, MA, USA). The detection limits of assay were between 24.69 pg/ml and 6000 pg/ml.

Definition of metabolic syndrome, hyperuricemia and subclinical hypothyroidism.

The metabolic syndrome (MetS) was defined by three out of the following five components: WC ≥ 94 cm in males and ≥80 cm in females, hypertriglyceridemia (≥1.7 mmol/l), low HDL-C (< 1.0 mmol/l in males or <1.3 mmol/l in females), increased BP (SP ≥ 130 mmHg and/or DP ≥ 85 mmHg and/) or positive history of hypertension or on any hypertensive treatment, FPG ≥ 5.6 mmol/l or on any hypoglycemic drugs [14]. Hyperuricemia was defined as serum UA ≥ 7 mg/dl (≥417 umol/L) in males, and ≥ 6 mg/dl (≥357 umol/L) in females [15]. Mild increased TSH was defined in this study as a TSH level ranged from 2.5 to 5.5 mU/l and a normal free-thyroxine level [16, 17].

## Statistical analysis

All data were performed using SPSS 17.0 software. All continuous values were presented as means ± standard deviation($\overline{X}$±s) and the count data were expressed as the number of columns (n). Quantitative data were analyzed using a t-test between two groups. The correlation between BMP-4 and other variables were assessed using Pearson’s correlation analysis. Multivariate regression analysis was performed to determine independent contributing factors for BMP-4 levels. Non-normally distributed variables were logarithmically transformed before analysis. Statistical differences were two tailed with *P* value less than 0.05.

## Results

### Demographic and biochemical parameters

Table 1 shows clinical characteristics and biochemical data for the 69 obese subjects with BMI being 33.64 ± 5.17 kg/m^2^. Serum BPM-4 levels were 763.98 ± 324.11 pg/ml in all obese patients, and 727.83 ± 316.48 pg/ml in males and 813.85 ± 333.41 pg/ml in females respectively without significant difference. The NC, WHR, UA, FT3, FT4, %fat trunk/%fat legs were significantly higher in males than females (all *P* < 0.05). However, the HOMA-IR, HDL-C, FFA, TSH, trunk fat%, Est. VAT Area, total fat% and trunk fat mass were significantly higher in females than males (all *P* < 0.05). There were no statistical differences of other parameters between males and females.

**Table 1:** Clinical and biochemical characteristics of the obese patients

Parameters	All patients (*N* = 69)	Males (*N* = 39)	Females (*N* = 30)
Years old	43.71 ± 12.72	44.05 ± 13.19	43.25 ± 12.47
BMP-4(pg/mL)	763.98 ± 324.11	727.83 ± 316.48	813.85 ± 333.41
BMI(kg/m^2^)	33.64 ± 5.17	32.69 ± 5.12	34.91 ± 5.04
Weight(kg)	96.16 ± 15.80	98.36 ± 15.11	93.20 ± 16.49
NC(cm)	40.56 ± 3.61	41.97 ± 2.72*	38.64 ± 3.83
WC(cm)	107.21 ± 9.64	107.08 ± 9.17	107.40 ± 10.51
HC(cm)	110.46 ± 10.76	108.80 ± 9.09	112.80 ± 12.55
WHR	0.97 ± 0.05	0.98 ± 0.05*	0.95 ± 0.05
SBP(mmHg)	136.86 ± 15.10	139.11 ± 13.58	133.78 ± 16.85
DBP(mmHg)	85.22 ± 10.16	86.19 ± 10.01	83.89 ± 10.50
ALT(U/L)	34.37 ± 14.13	34.24 ± 12.43	34.53 ± 16.18
AST(U/L)	25.64 ± 9.89	24.79 ± 9.33	26.60 ± 10.56
FPG(mmol/L)	8.60 ± 3.78	8.06 ± 3.86	9.24 ± 3.63
FINS(uU/mL)	25.63 ± 13.19	23.10 ± 11.43	29.28 ± 14.87
C-peptide(ng/mL)	3.71 ± 1.19	3.66 ± 1.40	3.78 ± 0.84
HOMA-IR	10.09 ± 9.79	7.65 ± 5.06*	13.26 ± 13.16
HbA1C %	7.27 ± 1.56	7.23 ± 1.70	7.32 ± 1.40
TC(mmol/L)	5.66 ± 1.13	5.68 ± 1.14	5.62 ± 1.14
TG(mmol/L)	3.05 ± 2.68	3.42 ± 3.08	2.57 ± 2.02
HDL-C(mmol/L)	1.17 ± 0.27	1.06 ± 0.18**	1.31 ± 0.30
LDL-C(mmol/L)	3.30 ± 0.90	3.34 ± 0.76	3.24 ± 1.06
CRP(mg/L)	3.47 ± 4.11	2.72 ± 3.20	4.58 ± 5.03
FFA(mmol/L)	0.57 ± 0.18	0.53 ± 0.17*	0.64 ± 0.17
UA(umol/L)	403.14 ± 100.54	446.94 ± 95.43**	348.75 ± 78.68
FT3(pmol/L)	4.87 ± 0.51	5.10 ± 0.49**	4.56 ± 0.36
FT4(pmol/L)	16.00 ± 2.13	16.64 ± 2.18*	15.13 ± 1.75
TSH(mU/L)	2.03 ± 0.85	1.80 ± 0.74*	2.35 ± 0.90
%fat trunk/%fat Legs	1.29 ± 0.16	1.35 ± 0.12*	1.19 ± 0.16
Total fatmass(kg)	95.60 ± 15.27	98.07 ± 14.53	92.03 ± 16.02
Trunkfat%	41.49 ± 5.61	38.41 ± 4.62**	44.95 ± 3.54
Est. VAT Area(cm^2^)	226.07 ± 50.38	210.71 ± 44.20*	247.76 ± 51.80
Total fat%	38.11 ± 6.31	34.41 ± 4.62**	43.45 ± 4.25
Trunk fatmass(g)	20.30 ± 4.84	19.04 ± 4.68*	22.06 ± 4.64
Trunk/limb fat mass ratio	1.45 ± 0.25	1.50 ± 0.24	1.36 ± 0.24

Student’s *t*-test was used. Compared to females, *P < 0.05, **P < 0.001

### Correlation of BMP-4 with anthropological and metabolic variables

The BMP-4 levels were 763.98 ± 324.11 pg/ml in all obese patients, and 727.83 ± 316.48 pg/ml in males and 813.85 ± 333.41 pg/ml in females respectively. In all subjects, BMP-4 levels were significantly positively associated with Est. VAT Area as presented in Table 2(all *P* < 0.05). Correlation of BMP-4 with fat distribution showed that BMP-4 levels were also significantly positively associated with Est. VAT Area and total fat% in females as shown in Table 2(all *P* < 0.05). Additionally, BMP-4 was also significantly negatively correlated with TC in all subjects and males (all *P* < 0.05). BMP-4 was also significantly positively associated with FT3 in males (*P* < 0.05). In Table 3, the multiple linear regression analysis results showed that Est. VAT Area was independently related to BMP-4 when other potential confounding variables were included in the female group. However, in all subjects and males, TC was the influencing factor of serum BMP-4 concentration.

**Table 2:** Correlations of serum BMP-4 levels with anthropometric variables, glucose-lipid metabolism and fat content

Parameters	Male		Female		Total	
	r	*P*	r	*P*	r	*P*
BMI	0.032	0.848	0.375	0.045	0.201	0.100
Weight	0.118	0.476	0.365	0.052	0.201	0.100
NC	0.068	0.702	−0.145	0.490	−0.096	0.468
WC	0.136	0.436	0.233	0.263	0.181	0.166
lnHC	−0.001	0.995	0.429	0.029	0.225	0.083
lnWHR	0.206	0.236	−0.416	0.035	−0.097	0.462
SBP	-0.173	0.397	0.244	0.314	−0.006	0.970
DBP	-0.126	0.541	0.251	0.301	0.020	0.894
ALT	0.069	0.694	−0.072	0.712	−0.003	0.982
AST	0.203	0.256	0.080	0.678	0.152	0.237
FPG	-0.095	0.588	0.376	0.147	0.089	0.486
FINS	-0.132	0.422	−0.026	0.896	0.152	0.217
C-peptide	−0.032	0.847	−0.290	0.127	−0.103	0.404
lnHOMA-IR	-0.196	0.258	0.214	0.284	−0.077	0.550
HbA1C	−0.113	0.510	0.161	0.404	0.001	0.994
TC	-0.405	0.013	−0.332	0.079	−0.373	0.002
lnTG	−0.244	0.146	−0.193	0.308	−0.231	0.062
HDL-C	-0.166	0.326	−0.025	0.897	−0.022	0.858
LDL-C	-0.231	0.168	−0.158	0.412	−0.195	0.116
lnCRP	−0.131	0.431	0.019	0.924	−0.098	0.441
FFA	-0.080	0.638	0.186	0.335	0.069	0.582
UA	0.032	0.851	0.209	0.276	0.039	0.759
FT3	0.441	0.005	−0.151	0.434	0.124	0.314
FT4	0.045	0.786	−0.150	0.436	−0.072	0.558
TSH	0.100	0.547	0.214	0.266	0.186	0.129
%fat trunk/%fat legs	0.002	0.993	−0.358	0.144	−0.221	0.149
Total fatmass	−.034	0.870	0.445	0.064	0.161	0.298
Trunkfat%	−0.304	0.131	0.444	0.065	0.055	0.725
Est.VAT Area	0.128	0.552	0.625	0.007	0.377	0.015
Total Fat%	−0.236	0.247	0.493	0.038	0.139	0.370
Trunk fatmass	−0.100	0.635	0.395	0.105	0.170	0.275
Trunk/limb fat mass ratio	0.053	0.795	−0.370	0.131	−0.164	0.287

**Table 3:** Multivariate analysis for risk factors of BMP-4

	Constant	*β*	Sd.E	*t*	Sig
Male	FT3	22.705	75.348	0.301	0.764
	TC	-105.043	34.877	−3.012	0.004
Female	Est.VAT Area	6.887	3.029	2.273	0.044
	Total fat%	−14.773	35.644	−0.414	0.686
	lnHC	−957.620	1422.012	−0.673	0.515
	lnWHR	−2301.084	1481.105	−1.554	0.149

### Difference of BMP-4 levels between obesity related diseases

**Fig. 1:**
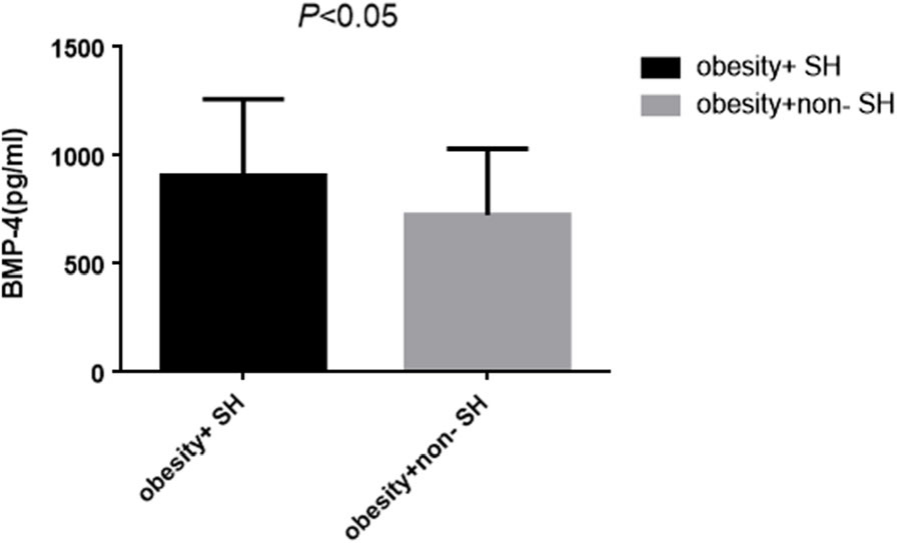
BMP-4 levels in obesity with mild increased TSH and without mild increased TSH

**Fig. 2:**
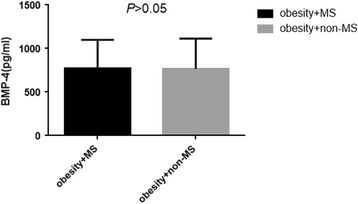
BMP-4 levels in obesity with Mets and without Mets

**Fig. 3:**
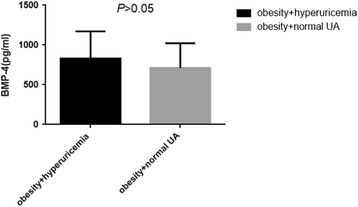
BMP-4 levels in obesity with hyperuricemia and without hyperuricemia

In these obese subjects, the prevalence of Mets, hyperuricemia and slightly increased TSH accounted for 62.31%, 52.17% and 26.08% respectively. BMP-4 levels were significantly higher in obesity with slight increase in TSH than obesity without slight increase in TSH (902.08 ± 354.74 pg/ml vs. 720.24 ± 306.41 pg/ml, *P* < 0.05) as presented in Fig. 1. Meanwhile, BMP-4 levels were slight higher in those with both Mets and hyperuricemia when compared to those without the corresponding complications (773.39 ± 325.79 pg/ml vs. 767.36 ± 347.20 pg/ml; 831.26 ± 340.72 pg/ml vs. 712.23 ± 309.99 pg/ml, all *P* > 0.05) as shown in Figs. 2 and 3.

### Change in BMP-4 and other parameters of Exenatide treatment

**Fig. 4:**
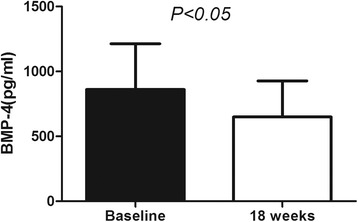
Change of BMP-4 levels after Exenatide treatment

After 18 weeks of Exenatide treatment, the BMP-4 levels in 30 obese patients were significantly decreased from 860.05 ± 352.65 pg/ml to 649.44 + 277.49 pg/ml(*P* = 0.01), shown in Fig. 4. The body weight and BMI were also significantly decreased from 94.86 ± 15.70 to 93.25 ± 16.37 kg(*P* = 0.024) and 33.52 ± 5.03 to 32.88 ± 5.42 kg/m^2^(*P* = 0.01). However, the change of BMP-4 was not significantly associated with the change in body weight and BMI (*P* > 0.05). Additionally, following Exenatide intervention, the glucose metabolism was significantly improved, shown in Table 4.

**Table 4:** Change of glucose metabolism after Exenatide intervention

	Baseline	After	*P*- value
FPG(mmol/l)	9.58 ± 3.86	7.36 ± 2.41	<0.001
FINS (uU/l)	28.30 ± 14.17	22.53 ± 10.47	0.007
HOMA-IR	12.84 ± 13.01	7.61 ± 5.54	0.013
HbA1C (%)	7.79 ± 1.72	6.50 ± 0.96	<0.001

## Discussion

Obesity is a worldwide health problem which need intervention and the patient may be the most important member of the health management [18]. Excess fat deposition is the manifestation of obesity. The ability of storing excess energy in adipose tissue is an evolutionary adaptation. However, the excessive fat mass accumulation gives rise to insulin resistance and inflammatory response [19]. Affected patients are often insulin resistant and dyslipidemic. It is also a high risk factor for metabolic syndrome. BMPs are members of the transforming growth factor β superfamily [20]. BMP-4 secreted by white adipose cells has been implicated in regulating adipogenic precursor cell commitment and differentiation. Differentiated human adipose cells can promote adipogenesis via endogenous BMP-4 activation [6]. BMP-4 antagonist inhibits the differentiation of pre-adipocytes into adipocytes [21]. BMP-4 also has an important role in promoting brown adipocyte differentiation and thermogenesis in vivo [22]. BMP-4 alters insulin sensitively by changing white adipose tissue [23]. A study also found that BMP-4 levels are significantly higher in obesity patients with metabolic syndrome [11]. However, little study has investigated the association of BMP-4 levels with fat distribution in obesity. Additionally, the relationship between GLP-1 treatment and BMP-4 levels were unclear. The aim of the current study was to investigate the serum BMP-4 levels in relation to fat distribution in obese patients and to examine changes brought about by Exenatide treatment. This study suggested that serum BPM-4 levels were significantly positively correlated with Est. VAT Area and negatively correlated with TC in all subject. Serum BPM-4 levels were significantly positively correlated with FT3 and negatively correlated with TC in males. The serum BMP-4 levels were significantly positively correlated with BMI, Est. VAT Area and total fat% in females. Among these parameters, TC was the influencing factor of serum BMP-4 concentration in all subjects and males, and Est. VAT area was the independent contributory factor to serum BMP-4 concentration in female. After 18 weeks Exenatide treatment, the BMP-4 levels were significantly decreased.

Animal study has suggested that BMP-4 mRNA expression was increased in inguinal fat of ob/ob mice when compared to control lean mice [1]. Serum BMP-4 levels were positively associated with BMI in human [11]. Our study suggested that the serum BMP-4 levels were also significantly positively correlated with BMI in females with obesity.

The adipose tissue of obesity is the foundation of metabolic disease [24]. Fat deposition in different part of the body has different degree of metabolic risk although the same amount of total fat mass may be unchanged [25]. Nonalcoholic fatty liver(NAFLD) caused by abnormal hepatic fat deposition is due to more than just impaired adenosine-monophosphate-activated protein kinase (AMPK)-peroxisome proliferator-activated receptor alpha(PPAR-α) signaling [26]. The ectopic fat accumulation also has a range of metabolic and cardiovascular effects. Central obesity is high risk factor of T2DM [27–29]. WHR and WC are the indicators of central obesity [30]. Study has found that serum BMP-4 levels were positively associated with WC and WHR [11]. As excess abdominal visceral fat is strongly associated with abnormal metabolic profile, we further researched the association of BMP-4 levels with fat content and its distribution. The results showed that the BMP-4 levels were significantly positively related to the trunk fat mass, especially visceral adipose tissue in the current study in total subjects and females. BMP-4 levels were also significantly positively related to total fat% and HC in female and negatively related to WHR in females. However, the association in males was not significant. This may due to the WHR and NC were significantly higher in males than females while the visceral adipose tissue was significantly higher in females than males. Multivariate analyses also reminded that the Est. VAT area was the risk factor for serum BMP-4 concentration in females. Therefore, we infer that visceral adipose tissue may play an important role in BMP-4 levels in females.

On the relationship between BMP-4 and glucose-lipid metabolic disorders in obese patients, a previous study has shown that serum BMP-4 levels were positively correlated with fasting plasma insulin, homeostasis model assessment index, and triglycerides and negatively correlated with HDL-C in obese individuals [11]. However, in this study, BMP-4 levels were negatively associated with TC levels in all subjects, especially in male. Visceral fat accumulation results in the obesity-associated dyslipidemia [31]. However, TC is significantly associated with the ectopic visceral fat and subcutaneous fat in women not man [32]. Therefore, the association between BMP-4 and TC levels in males may be due to the visceral fat accumulation, which is slightly less in males than females in our study. Previous study has showed that serum BMP-4 levels are significantly positively associated with adiposity, insulin resistance [11]. However, other study showed that BMP-4 expression is up-regulated in human adipocytes to play a protective anti-inflammatory effect [33]. BMP-4 acts as an anti-inflammatory molecule via increasing proliferator-activated receptor γ (PPARγ) expression and reducing pro-inflammatory cytokines tumor necrosis factor α (TNF-α) in human adipocytes [33]. BMP-4 may be a protective factor in males. There need more studies to prove and explain it.

Previous study has shown that subclinical hypothyroidism(SH), characterized by mildly increased TSH, is a secondary phenomenon of the abnormal fat accumulation and redistributions, and not a real hypothyroid state in obesity [34]. Studies also indicate that obesity is a risk factor for hyperuricemia and hyperuricemia may be affected by body fat distribution [35, 36]. Obesity is one of the components of metabolic syndrome and previous study has shown that serum BMP-4 levels were significantly increased in individuals with obesity or metabolic syndrome [11]. Therefore, in the current study, we compared the BMP-4 levels in obesity associated with different types of metabolic disorder to obesity without the corresponding complications. The results showed that BMP-4 levels were significantly higher in obesity with slight increased TSH than without increased TSH. Central obesity is associated with SH, especially the visceral fat deposition is related to increased TSH [37, 38]. Therefore, it may be the increased TSH with more serious visceral fat deposition that caused the higher BMP-4 levels in this study. Besides, BMP-4 levels were slightly higher in obesity with Mets or hyperuricemia. However, the difference has no statistical significance. It may be due to the limit sample.

GLP-1 receptor agonist is useful in treating diabetes. It is also efficient in decreasing weight loss in the obesity by restraining appetite. Exenatide is a GLP-1 receptor agonist that has been used in clinic. In our study, 18 weeks Exenatide treatment led to a significant decrease in BMP-4 levels, independent of weight loss. Therefore, we infer that there may exist interaction between GLP-1 and BMP-4. We deduce the metabolic improvement by weight loss and fat distribution treated by exenatide may lead to the decreased BMP-4 levels. We will design the animal experiment to elucidate the underlying mechanism.

There also exist limitations in this study. Primarily, our study was limited because of the relatively small number of patients. Secondly, our results might not be universal because subjects enrolled were only Chinses, therefore, it may not be applied to other races. Finally, it is a mechanism which has yet to be fully elucidated. Therefore, more studies with larger sample are required to confirm the results. More basic studies are warranted to expound the signaling mechanisms linking BMP4 with fat distribution.

## Conclusions

In summary, this was the first study that demonstrated that serum BMP-4 levels were associated with visceral fat content in obese patients. The role of BMP-4 in males and females may be different. Visceral adipose mass may predict the serum BMP-4 levels in females with obesity. BMP-4 were significantly decreased after Exenatide treatment in obesity. Therefore, further studies are required to investigate the relationship between serum BMP4 levels and the pathogenesis of obesity.

## Abbreviations

ALT: Alanine transaminase
AMPK: Monophosphate-activated protein kinase
AST: Aspartate aminotransferase
BAT: Brown adipose tissue
BMP-4: Bone morphogenetic protein 4
BP: Blood pressure
CP: C-peptide
CRP: C reactive protein
DEXA: Dual-energy X-ray absorptiometry
DP: Diastolic pressure
Est. VAT Area: Estimated visceral adipose tissue Area
FFA: Free fatty acids
FINS: Fasting insulin
FPG: Fasting plasma glucose
FT3: Free triiodothyronine
FT4: Free thyroxine
GLP-1: Glucagon-like peptide-1
HbA1C: Hemoglobin A1C
HC: Hip circumference
HDL-C: High density lipoprotein cholesterol
HOMA-IR: Homoeostasis Model of Insulin Resistance
LDL-C: Low density lipoprotein cholesterol
MetS: Metabolic syndrome
NC: Neck circumference
PPARγ: Proliferator-activated receptor γ
SH: Subclinical hypothyroidism
SP: Systolic pressure
T2DM: Type-2 diabetes
TC: Total cholesterol
TG: Triglyceride
TNF-α: Tumor necrosis factor α
TSH: Thyroid stimulating hormone
UA: Uric acid
WAT: White adipose tissue
WC: Waist circumference
WHR: Waist to hip ratio

## References

[1] Bowers RR, Lane MD (2007). A role for bone morphogenetic protein-4 in adipocyte development[J]. Cell Cycle (Georgetown, Tex).

[2] Konieczny SF, Emerson CP (1984). 5-Azacytidine induction of stable mesodermal stem cell lineages from 10T1/2 cells: evidence for regulatory genes controlling determination[J]. Cell.

[3] Spiegelman BM, Flier JS (2001). Obesity and the regulation of energy balance[J]. Cell.

[4] Neeland IJ, Turer AT, Ayers CR (2012). Dysfunctional adiposity and the risk of prediabetes and type 2 diabetes in obese adults[J]. JAMA.

[5] Gastaldelli A, Harrison SA, Belfort-Aguilar R (2009). Importance of changes in adipose tissue insulin resistance to histological response during thiazolidinedione treatment of patients with nonalcoholic steatohepatitis[J]. Hepatology (Baltimore, Md).

[6] Gustafson B, Smith U (2012). The WNT inhibitor Dickkopf 1 and bone morphogenetic protein 4 rescue adipogenesis in hypertrophic obesity in humans[J]. Diabetes.

[7] Gustafson B, Hammarstedt A, Hedjazifar S (2013). Restricted adipogenesis in hypertrophic obesity: the role of WISP2, WNT, and BMP4[J]. Diabetes.

[8] Bowers RR, Kim JW, Otto TC (2006). Stable stem cell commitment to the adipocyte lineage by inhibition of DNA methylation: role of the BMP-4 gene[J]. Proc Natl Acad Sci U S A.

[9] Gustafson B, Hammarstedt A, Hedjazifar S (2015). BMP4 and BMP Antagonists Regulate Human White and Beige Adipogenesis[J]. Diabetes.

[10] Matsuzawa Y, Funahashi T, Nakamura T (1999). Molecular mechanism of metabolic syndrome X: contribution of adipocytokines adipocyte-derived bioactive substances[J]. Ann N Y Acad Sci.

[11] Son JW, Kim MK, Park YM (2011). Association of serum bone morphogenetic protein 4 levels with obesity and metabolic syndrome in non-diabetic individuals[J]. Endocr J.

[12] Lockie SH, Heppner KM, Chaudhary N (2012). Direct control of brown adipose tissue thermogenesis by central nervous system glucagon-like peptide-1 receptor signaling[J]. Diabetes.

[13] Wallace TM, Levy JC, Matthews DR (2004). Use and abuse of HOMA modeling[J]. Diabetes Care.

[14] Alberti KG, Eckel RH, Grundy SM (2009). Harmonizing the metabolic syndrome: a joint interim statement of the International Diabetes Federation Task Force on Epidemiology and Prevention; National Heart, Lung, and Blood Institute; American Heart Association; World Heart Federation; International Atherosclerosis Society; and International Association for the Study of Obesity[J]. Circulation.

[15] Ryu KA, Kang HH, Kim SY (2014). Comparison of nutrient intake and diet quality between hyperuricemia subjects and controls in Korea[J]. Clin Nutr Res.

[16] Spencer CA, Schwarzbein D, Guttler RB (1993). Thyrotropin (TSH)-releasing hormone stimulation test responses employing third and fourth generation TSH assays[J]. J Clin Endocrinol Metab.

[17] Vanderpump MP, Tunbridge WM, French JM (1995). The incidence of thyroid disorders in the community: a twenty-year follow-up of the Whickham Survey[J]. Clin Endocrinol.

[18] Ciccone MM, Aquilino A, Cortese F (2010). Feasibility and effectiveness of a disease and care management model in the primary health care system for patients with heart failure and diabetes (Project Leonardo)[J]. Vasc Health Risk Manag.

[19] Haslam DW, James WP (2005). Obesity[J]. Lancet.

[20] Massague J, Chen YG (2000). Controlling TGF-beta signaling[J]. Genes Dev.

[21] Huang H, Song TJ, Li X (2009). BMP signaling pathway is required for commitment of C3H10T1/2 pluripotent stem cells to the adipocyte lineage[J]. Proc Natl Acad Sci U S A.

[22] Xue R, Wan Y, Zhang S (2014). Role of bone morphogenetic protein 4 in the differentiation of brown fat-like adipocytes[J]. Am J Phys Endocrinol Metab.

[23] Qian SW, Tang Y, Li X (2013). BMP4-mediated brown fat-like changes in white adipose tissue alter glucose and energy homeostasis[J]. Proc Natl Acad Sci U S A.

[24] Mohamed-Ali V, Pinkney JH, Coppack SW (1998). Adipose tissue as an endocrine and paracrine organ[J]. Int. J. Obes. Relat. Metab. Disord..

[25] Despres JP, Moorjani S, Lupien PJ, et al. Regional distribution of body fat, plasma lipoproteins, and cardiovascular disease[J]. Arteriosclerosis (Dallas, Tex), 1990,10(4):497-511. 10.1161/01.atv.10.4.497 2196040

[26] Gu Q, Yang X, Lin L (2014). Genetic ablation of solute carrier family 7a3a leads to hepatic steatosis in zebrafish during fasting[J]. Hepatology.

[27] Despres JP, Lemieux I (2006). Abdominal obesity and metabolic syndrome[J]. Nature.

[28] Carey VJ, Walters EE, Colditz GA (1997). Body fat distribution and risk of non-insulin-dependent diabetes mellitus in women. The Nurses' Health Study[J]. Am J Epidemiol.

[29] Fox KA, Despres JP, Richard AJ (2009). Does abdominal obesity have a similar impact on cardiovascular disease and diabetes? A study of 91,246 ambulant patients in 27 European countries[J]. Eur Heart J.

[30] Martins IS, Marinho SP. [The potential of central obesity anthropometric indicators as diagnostic tools]. Rev Saude Publica 2003,37(6):760-767. 10.1590/s0034-89102003000600011 14666306

[31] Kihara S. [Dyslipidemia][J]. Nihon Rinsho Japanese J Clin Med, 2013,71(2):275-279. 23631206

[32] Roever LS, Resende ES, Diniz AL (2016). Abdominal Obesity and Association With Atherosclerosis Risk Factors: The Uberlandia Heart Study[J]. Medicine.

[33] Baraban E, Chavakis T, Hamilton BS (2016). Anti-inflammatory properties of bone morphogenetic protein 4 in human adipocytes[J]. Int J Obes (2005).

[34] Ruiz-Tovar J, Boix E, Galindo I (2014). Evolution of subclinical hypothyroidism and its relation with glucose and triglycerides levels in morbidly obese patients after undergoing sleeve gastrectomy as bariatric procedure[J]. Obes Surg.

[35] Matsuura F, Yamashita S, Nakamura T (1998). Effect of visceral fat accumulation on uric acid metabolism in male obese subjects: visceral fat obesity is linked more closely to overproduction of uric acid than subcutaneous fat obesity[J]. Metab Clin Exp.

[36] Furukawa N, Ongusaha P, Jahng WJ (2005). Role of Rho-kinase in regulation of insulin action and glucose homeostasis[J]. Cell Metab.

[37] Sieminska L, Wojciechowska C, Walczak K (2015). Associations between metabolic syndrome, serum thyrotropin, and thyroid antibodies status in postmenopausal women, and the role of interleukin-6[J]. Endokrynologia Polska.

[38] Giandalia A, Russo GT, Romeo EL (2014). Influence of high-normal serum TSH levels on major cardiovascular risk factors and Visceral Adiposity Index in euthyroid type 2 diabetic subjects[J]. Endocrine.

